# Anesthetic challenges in a child with Alport syndrome undergoing prone surgery with a laryngeal mask airway: a case report

**DOI:** 10.3389/fped.2026.1950654

**Published:** 2026-10-21

**Authors:** Christine Gaik, Selena Knoth, Felix Schmitt, Ingelore Holzinger

**Affiliations:** 1 Philipps-Universität Marburg, Marburg, Germany; 2 Department of Anesthesiology and Operative Intensive Care Medicine, University Hospital Giessen and Marburg, Campus Marburg, Marburg, Germany; 3 Department of Pediatric Anesthesia and Intensive Care Medicine, Klinikum Kassel, Kassel, Germany

**Keywords:** Alport syndrome, case report, laryngeal mask airway, pediatric anesthesia, prone position, rare disease

## Abstract

**Introduction:**

Alport syndrome is a rare hereditary disorder caused by type IV collagen abnormalities and characterized by progressive kidney disease with variable extrarenal manifestations. Evidence guiding anesthetic management, particularly in pediatric patients, remains limited.

**Case presentation:**

We report the anesthetic management of a 12-year-old girl with a clinical diagnosis of X-linked Alport syndrome based on microscopic hematuria and obligate heterozygosity for a familial pathogenic COL4A5 variant undergoing elective lower-leg surgery in the prone position. Despite preserved renal function, perioperative management included careful drug selection and a propofol-based total intravenous anesthesia using a second-generation laryngeal mask airway without neuromuscular blockade. Stable ventilation was maintained throughout prone surgery, and recovery was uncomplicated with effective postoperative pain control.

**Conclusions:**

This case contributes to the limited literature on anesthetic management in pediatric Alport syndrome and highlights practical perioperative considerations regarding airway management, anesthetic technique, and renal-protective care.

## Introduction

First described in 1927 by Arthur Cecil Alport in a family with persistent hematuria and progressive hearing loss, Alport syndrome is a hereditary multisystem disorder caused by mutations affecting the *α*3, *α*4, and *α*5 chains of type IV collagen, a major structural component of the glomerular basement membrane (1–4). The disease is classically characterized by progressive renal impairment, sensorineural hearing loss, and ocular abnormalities (5). Owing to its systemic nature, particularly renal dysfunction and potential extrarenal manifestations, Alport syndrome presents specific challenges for anesthetic management and perioperative care.

We report the anesthetic management of a child with X-linked Alport syndrome undergoing lower-limb surgery in the prone position using propofol-based total intravenous anesthesia (TIVA) and a second-generation laryngeal mask airway (LMA). This case highlights practical perioperative considerations regarding anesthetic technique, airway management, perioperative monitoring, and renal-protective drug selection in this rare hereditary disorder.

## Case report

### Past medical history

The patient is a 12-year-old girl (height 154 cm, weight 63 kg, BMI 26.6 kg/m^2^) with a clinical diagnosis of X-linked Alport syndrome based on microscopic hematuria and her status as an obligate heterozygote for a familial pathogenic COL4A5 variant. The patient did not pass the newborn hearing screening. However, comprehensive audiological evaluation subsequently demonstrated normal hearing, which remained unchanged on repeated follow-up assessments performed because of the positive family history. Motor and language development were age-appropriate.

Alport syndrome first became clinically apparent four years before the current presentation, when isolated microscopic hematuria was incidentally detected during the diagnostic work-up following a bicycle accident with blunt abdominal trauma caused by the handlebar. Her father had previously received a molecular genetic diagnosis of X-linked Alport syndrome. Following genetic counselling, direct molecular genetic testing of the patient was not performed. As all daughters of affected males inherit the paternal X chromosome carrying the familial pathogenic COL4A5 variant, the patient was considered an obligate heterozygote. To date, she has shown no evidence of renal insufficiency, hearing impairment, or ocular involvement.

The family history demonstrated marked phenotypic variability. The patient's father developed childhood-onset cataracts and progressive hearing loss requiring hearing aids. At the age of 25 years, he developed end-stage kidney disease requiring dialysis and subsequently underwent kidney transplantation. Molecular genetic testing in the father identified the pathogenic COL4A5 frameshift variant, c.1989delG (p.K664Sfs*14), which was also present in the paternal grandmother, who had mild renal impairment with proteinuria and hematuria. The patient's younger sister (8 years old) carried the same familial variant but remained clinically asymptomatic.

The patient was born after an uneventful pregnancy at 38 + 1 weeks of gestation with a birth weight of 2,800 g.

### Allergies, comorbidities and medication

Comorbidities included exercise-induced bronchial asthma, treated with salbutamol as needed. Following the incidental detection of microscopic hematuria four years earlier and the subsequent diagnosis of X-linked Alport syndrome, ramipril (5 mg once daily in the evening) had been prescribed as long-term therapy and was continued throughout the perioperative period. No allergies were reported.

### Previous anesthetic history

Her anesthetic history included a general anesthetic in August 2021 for resection of a vascular malformation of the left calf using a supraglottic airway. In December 2025, she underwent general anesthesia for strabismus surgery at an external institution, which was complicated by postoperative nausea and vomiting (PONV) (Table 1).

**Table 1:** Timeline of the patient's clinical history, diagnosis, and perioperative management.

Time	Clinical event
Neonatal period	Failed newborn hearing screening; subsequent audiological assessments remained normal.
August 2021	Resection of a venous malformation of the left calf under general anesthesia using a supraglottic airway.
2022	Incidental detection of microscopic hematuria following blunt abdominal trauma. Clinical diagnosis of X-linked Alport syndrome based on family history and initiation of ramipril therapy.
May 2025	Recurrence of the venous malformation with exertional pain and bluish swelling at the previous surgical site.
December 2025	Strabismus surgery under general anesthesia complicated by PONV.
January 2026	Preoperative evaluation demonstrated preserved renal function and no evidence of hearing or ocular involvement.
January 2026	Elective resection of the recurrent venous malformation in the prone position under propofol-based TIVA using a second-generation laryngeal mask airway. Intraoperative course was uneventful.
POD 2	Discharged home following an uncomplicated postoperative recovery.
POD 8	Outpatient follow-up showed satisfactory wound healing without signs of infection.

POD, Postoperative day; PONV, Postoperative nausea and vomiting; TIVA, Total intravenous anesthesia.

### Current medical history

The patient was scheduled for elective resection of a recurrent venous malformation of the left lower leg in January 2026. Approximately four years after the initial resection, she developed recurrent exertional pain and bluish swelling at the previous surgical site, first noted in May 2025.

### Preoperative anesthetic assessment

Preoperative evaluation revealed a patient in good general condition with normal exercise tolerance. Airway assessment showed stable dentition, an upper jaw orthodontic appliance, a mandibular retainer, and a Mallampati score of I. No predictors of difficult mask ventilation or airway management were identified. The patient was classified as ASA physical status II. During preoperative consent, the patient and her mother were informed about general anesthesia using either a supraglottic airway device or an endotracheal tube. Routine laboratory testing performed one month before surgery was within normal limits, including a serum creatinine level of 0.64 mg/dL (reference range 0.26–0.77 mg/dL), a serum urea level of 32 mg/dL (reference range 15–36 mg/dL), and a serum potassium concentration of 4.1 mmol/L (reference range 3.3–4.7 mmol/L). Cystatin C was not assessed as part of the current preoperative evaluation; however, a measurement obtained one year earlier was within the reference range (1.0 mg/L; reference range 0.59–1.49 mg/L). An estimated glomerular filtration rate was not determined, and no perioperative urinalysis or assessment of proteinuria or albuminuria was performed.

As a peripheral intravenous cannula (20-gauge) had already been placed on the ward, no additional oral or intranasal premedication was required.

### Anesthetic management

On arrival in the anesthesia preparation area, peripheral oxygen saturation (SpO_2_) was 99% on room air, blood pressure was 120/70 mmHg, and ECG showed sinus rhythm at 100 beats/min. After standard monitoring and preoxygenation, anesthesia was induced with fentanyl 150 μg (2.38 μg/kg) and propofol 150 mg (2.38 mg/kg). Airway management and maintenance of anesthesia were primarily performed by a consultant pediatric anesthesiologist with extensive experience in pediatric anesthesia. Mask ventilation was uneventful, and a second-generation LMA (LMA Supreme™, Teleflex), size 4, selected according to the manufacturer's weight-based sizing recommendation (50–70 kg), was inserted without difficulty. Cuff pressure was adjusted to 55 cmH₂O, achieving an effective seal. A gastric tube was inserted through the drainage channel, allowing aspiration of residual gastric contents.

After induction, a second peripheral venous access (20-gauge) was established. Anesthesia was maintained with propofol at 6.35 mg/kg/h (106 µg/kg/min), titrated according to bispectral index monitoring (BIS values 41–61), and remifentanil at 15.9 µg/kg/h (0.265 µg/kg/min). An additional bolus of fentanyl 50 μg (0.79 μg/kg) was administered intraoperatively. Before prone positioning, the surgical team was briefly informed about the use of the LMA and potential positioning-related complications. The patient was then turned from the supine to the prone position jointly by the surgical and anesthesia teams, with the anesthesia team maintaining manual stabilization of the head and secure fixation of the LMA throughout the maneuver. The head and neck were rotated laterally and supported on an open, crescent-shaped gel head ring, ensuring that the eyes and nose remained free from pressure. Particular care was taken to prevent contact between the LMA and the operating table, thereby avoiding external pressure on or displacement of the airway device; a flexible catheter mount allowed tension- and pressure-free positioning of the breathing circuit. Following repositioning, adequate LMA function and sealing were assessed by continuous monitoring of inspiratory and expiratory tidal volumes (VTi and VTe) and their difference (*Δ*VT), airway pressures, and ventilator leakage alarms, as well as by the absence of an audible leak. Cuff pressure was continuously monitored with an analog cuff pressure manometer and remained between 50 and 60 cmH₂O throughout surgery. The head position and airway device were regularly reassessed during the procedure. Mechanical ventilation was performed using pressure-controlled ventilation (PCV) with a PEEP of 5 cmH₂O, inspiratory pressures of 15–18 cmH₂O, VT of 6–8 mL/kg, minute ventilation of 6–7 L/min, and an FiO₂ between 0.41 and 0.48. End-tidal CO₂ (EtCO₂) was continuously monitored and ranged from 38 to 48 mmHg throughout the procedure, with ventilator settings adjusted as required to maintain adequate ventilation. From induction of anesthesia until removal of the LMA, SpO_2_ remained between 98% and 99%, sinus rhythm was maintained with a heart rate of 80–95 beats/min, and systolic blood pressure ranged from 95 to 110 mmHg, with MAP remaining ≥65 mmHg throughout without the need for vasoactive medication. Intraoperatively, approximately 300 mL of Ringer's acetate solution was administered intravenously. Blood loss was minimal. No urinary catheter was placed, and urine output was therefore not measured.

Perioperative antibiotic prophylaxis consisted of ampicillin/sulbactam 3 g (2 g/ 1 g; 47.6 mg/kg total dose) intravenously. Because of a previous episode of PONV, prophylaxis with dexamethasone 4 mg (63 μg/kg) and granisetron 1 mg (16 μg/kg) was administered. Co-analgesia was provided with dexmedetomidine 15 µg (0.24 μg/kg) intravenously and metamizole 1 g (15.9 mg/kg).

Temperature was measured nasally and maintained between 36.9 °C and 37.3 °C using forced-air warming.

A pneumatic thigh tourniquet was applied to the left leg by the surgical team and inflated to 200 mmHg for approximately 45 min to provide a bloodless surgical field. Immediately before tourniquet release, peripheral venous blood gas analysis was performed to establish baseline acid-base status and serum electrolyte values before reperfusion. PvCO₂ was 45 mmHg and showed no clinically relevant difference from the simultaneously measured EtCO₂; potassium and lactate concentrations were 3.9 mmol/L and 0.7 mmol/L, respectively. The surgical procedure was completed without complications. Total skin-to-skin time was 100 min. At the end of surgery, the patient was returned to the supine position. The LMA was removed after discontinuation of anesthesia once spontaneous ventilation had resumed with stable EtCO₂ values and spontaneous movements consistent with emergence from anesthesia were observed.

### Postoperative course and follow-up

Postoperatively, a Prelude brace was applied to the left thigh, and the patient was transferred to the post-anesthesia care unit (PACU) for approximately one hour. Vital signs remained stable throughout, and no supplemental oxygen was required. The modified Aldrete score was 12 on admission and 14 at discharge (6). In the PACU, postoperative pain reached a maximum visual analogue scale (VAS) score of 6 and improved following administration of 3 mg piritramide. No PONV, sore throat, or difficulty swallowing was observed.

She was subsequently transferred to the pediatric surgical ward. Pain was regularly assessed by nursing staff using the numeric rating scale (NRS), with documented scores ranging from 0 to 1. Metamizole 750 mg (11.9 mg/kg per dose) three times daily was administered according to a fixed schedule until discharge on postoperative day two and provided adequate baseline analgesia. A transient NRS score of 4 was documented once during physiotherapy and was adequately controlled following the scheduled metamizole dose. Postoperative mobilization with forearm crutches was uncomplicated and supported by physiotherapy. The subsequent course was uneventful, and the patient was discharged on postoperative day two. At an outpatient follow-up visit on day eight, the surgical wound showed no signs of irritation or infection.

## Discussion

This case describes the anesthetic management of a pediatric patient with Alport syndrome undergoing elective surgery. Although the patient presented with a clinically mild phenotype limited to isolated microscopic hematuria, Alport syndrome is a heterogeneous disorder with a potentially progressive course, as illustrated by the severe renal and extrarenal involvement in affected family members. From an anesthesiological perspective, this underlines the importance of a cautious perioperative approach even in the absence of overt organ dysfunction, particularly with regard to renal protection, drug selection, and overall perioperative management.

### Choice of anesthetic technique

In patients with Alport syndrome, renal involvement represents an important consideration for anesthetic management, even when renal function is clinically preserved. Although both inhalational anesthesia and TIVA are considered safe in patients without advanced renal impairment, careful drug selection and hemodynamic stability remain essential. In the present case, TIVA with propofol and remifentanil was chosen because of its predictable pharmacokinetics, favorable profile in a patient with a history of PONV, and the well-described effects of propofol on upper airway reflex responses (7–9). In addition, propofol-based TIVA has been associated with a lower risk of pediatric emergence delirium, whereas sevoflurane has been identified as a risk factor in a recent systematic review (10).

The use of a second-generation supraglottic airway allowed anesthesia without neuromuscular blockade. Consequently, no reversal agent was required, avoiding potential concerns related to the use of sugammadex in patients with advanced renal impairment. Although sugammadex is increasingly used in clinical practice in patients with severe renal impairment, its use in this population remains off-label because of limited safety data (11). In our patient, renal function was normal; nevertheless, minimizing unnecessary pharmacological interventions was considered appropriate in view of the lifelong risk of progressive renal involvement associated with COL4A5-related Alport syndrome (12). When tracheal intubation is required, remifentanil may be considered to facilitate intubation in selected patients in whom neuromuscular blocking agents are preferably avoided (13). If neuromuscular blockade is necessary in patients with impaired renal function, quantitative neuromuscular monitoring should be used to guide dosing and reversal (14).

Regional anesthesia may be feasible for lower-limb surgery but is commonly combined with general anesthesia in older children because anxiety, limited cooperation, and the risk of movement may compromise its use as the sole technique (15–18). The association of progressive sensorineural hearing loss in patients with Alport syndrome (19) should nevertheless be considered when planning communication and perioperative support, particularly in awake patients undergoing regional anesthesia.

In the present case, the prolonged prone position and anticipated tourniquet discomfort further favored general anesthesia, as tourniquet-related pain may still occur despite an otherwise adequate regional block, particularly during prolonged tourniquet inflation (20–22).

Both general anesthesia and neuraxial techniques have been reported in adults with Alport syndrome, although the latter are described only in isolated case reports of renal transplantation in patients with advanced renal disease and substantial comorbidity (23, 24).

Although evidence is limited to isolated case reports, type IV collagen abnormalities may theoretically increase the vulnerability of collagen IV-containing tissues beyond the kidney, including peripheral nerves and vascular or dural structures (25, 26). However, any implications for anesthetic management remain speculative and have not been systematically investigated.

In our patient, ramipril had been prescribed as long-term renoprotective therapy in the context of X-linked Alport syndrome rather than for hypertension or heart failure. Current perioperative recommendations for ACE inhibitors and angiotensin receptor blockers primarily address these latter indications (27, 28), whereas specific guidance for long-term renoprotective therapy is lacking. Given the limited surgical risk, short duration of the procedure, and anticipated minor volume shifts, continuation of ramipril was considered appropriate, consistent with national guidance allowing continuation irrespective of the indication in such settings (28).

### Airway management and prone positioning

Airway assessment in patients with Alport syndrome is generally similar to that of the general pediatric population, as the syndrome is not typically associated with craniofacial or upper airway abnormalities (19).

Rarely, Alport syndrome is associated with diffuse leiomyomatosis involving the esophagus and tracheobronchial tree (29–31). When mediastinal extension is present, airway management and patient positioning may require additional consideration because of the potential for compression of the tracheobronchial tree or major intrathoracic vessels (32).

Depending on the severity of renal involvement, gastroesophageal reflux may also warrant consideration during the preoperative assessment, as it has been reported in approximately 18% of patients with chronic kidney disease (33). However, our patient had preserved renal function, no history of reflux symptoms, and was appropriately fasted. Therefore, no apparent contraindications to the use of a second-generation supraglottic airway device were identified. Compared with first-generation devices, second-generation LMAs provide higher oropharyngeal leak pressures, allow gastric tube insertion and are therefore generally favored when a supraglottic airway is selected (34, 35). The choice of an LMA was primarily based on patient-specific considerations, with the patient's well-controlled exercise-induced asthma supporting its use over tracheal intubation to minimize airway stimulation (36). If tracheal intubation had become necessary, adequate anesthetic depth would have been ensured to minimize airway reactivity and the risk of bronchospasm. If bronchospasm had nevertheless occurred, management would have included further deepening anesthesia and inhaled salbutamol, with sevoflurane, intravenous magnesium, intravenous *β*₂-agonists, and neuromuscular blockade as additional options in severe cases (36). Neuromuscular blockade would also have been considered for persistent laryngospasm with inadequate ventilation.

Although the use of an LMA in the prone position in pediatric patients is not standard practice, elective and emergency applications in children have been described in case reports and a prospective clinical study (37–39). Survey data likewise suggest variation in clinical practice. In an international survey of anesthesiologists, 14% of respondents reported LMA use in both supine and prone positions, while a further 38.8% reported use in supine, lateral, and prone positions (40). In a survey of pediatric anesthesiologists, only 7.12% of respondents reported using an LMA for prone-position surgery (41). In the present case, the patient's normal airway anatomy, absence of reflux symptoms, low risk of aspiration, and the limited elective nature of the procedure supported the use of a securely fixed second-generation supraglottic airway, which allowed effective airway management while avoiding tracheal intubation and neuromuscular blockade. Ventilation followed lung-protective principles, providing stable ventilation and gas exchange throughout prone positioning. However, evidence defining optimal VT and PEEP in pediatric patients remains limited (42). Nevertheless, limited airway access in the prone position necessitates appropriate preparation for potential LMA failure. Accordingly, equipment for endotracheal intubation was kept immediately available, with a predefined rescue strategy involving interruption of surgery, return to the supine position, and subsequent tracheal intubation if adequate ventilation could not be maintained. The favorable outcome in this carefully selected patient supports the feasibility of this approach in selected cases but does not allow conclusions regarding its relative advantages over tracheal intubation in pediatric Alport syndrome.

### Perioperative pain management

Perioperative pain management in patients with Alport syndrome should consider the degree of renal involvement while incorporating nephroprotective principles, as preservation of kidney function is a key therapeutic objective in Alport syndrome (12). Although our patient had preserved renal function, a multimodal analgesic regimen was chosen with particular attention to avoiding potentially nephrotoxic medications, given the lifelong risk of progressive renal involvement (12). NSAIDs were avoided because they may impair renal function and increase the risk of acute kidney injury, particularly in susceptible patients (43, 44). Metamizole was therefore selected as the non-opioid analgesic. However, metamizole carries a potentially serious risk of agranulocytosis, which can occur independently of dose and should be considered in the individual risk-benefit assessment (45). Dexmedetomidine was added because of its analgesic and opioid-sparing effects in pediatric anesthesia (46, 47). An adjunct single-shot peripheral nerve block performed at the conclusion of surgery could have provided targeted, non-nephrotoxic analgesia while further reducing systemic analgesic requirements. Although this would have been a reasonable option, the decision not to perform a peripheral nerve block was based on an individual risk-benefit assessment considering the limited extent of the surgical procedure, the need for an additional intervention, and the potential for motor blockade. Intravenous multimodal analgesia provided adequate postoperative pain control with only a low opioid requirement in the PACU and an uncomplicated subsequent recovery.

### Perioperative monitoring

Perioperative monitoring should be adapted to the degree of renal involvement and the surgical procedure. In patients with advanced renal impairment, assessment of acid-base status and serum electrolytes, particularly potassium, may be warranted because of the risk of hyperkalemia (48). Tourniquet release may cause transient metabolic and electrolyte changes, although these are generally well tolerated in otherwise healthy patients (49). In our patient, venous blood sampling before tourniquet release documented normal baseline potassium and lactate concentrations before reperfusion.

Taken together, this case emphasizes that even children with clinically mild Alport syndrome may benefit from an individualized perioperative strategy that accounts for the disorder's potential progression and multisystem manifestations.

### Limitations

As a single case report, the findings are not generalizable. In particular, this patient's mild phenotype and preserved renal function may not reflect the perioperative challenges encountered in children with advanced Alport syndrome, including altered drug clearance, electrolyte disturbances, and hemodynamic instability. In addition, a patient perspective was not obtained as part of the clinical documentation and was therefore not available for this retrospective case report. Further clinical reports are needed to expand the evidence base.

## Conclusion

In this case of a child with a mild Alport phenotype and preserved renal function, propofol-based TIVA with a second-generation LMA was successfully used for prone lower-limb surgery. Perioperative management was guided by nephroprotective principles, careful drug selection, and awareness of the disorder's potential systemic manifestations and progressive nature. Because published anesthetic experience in pediatric Alport syndrome remains limited, this case highlights the importance of individualized perioperative planning based on the patient's current clinical status and potential for disease progression.

## Data Availability

The clinical data supporting the findings of this case report are included in the article. Additional patient-related data are not publicly available due to ethical and privacy restrictions. Requests to access the datasets should be directed to the corresponding author.
